# Work-related online learning during the COVID-19 pandemic in Germany

**DOI:** 10.1007/s40955-021-00192-5

**Published:** 2021-11-19

**Authors:** Corinna Kleinert, Gundula Zoch, Basha Vicari, Martin Ehlert

**Affiliations:** 1 Leibniz Institute for Educational Trajectories (LIfBi), Bamberg, Germany; 2 University of Bamberg, Bamberg, Germany; 3 University of Oldenburg, Oldenburg, Germany; 4 Institute for Employment Research (IAB), Nuremberg, Germany; 5 Berlin Social Science Center (WZB), Berlin, Germany

**Keywords:** Corona crisis, Adult education and training, Online learning, Working conditions, NEPS‑C, Corona Krise, Weiterbildung, Online-Lernen, Erwerbssituation, NEPS‑C

## Abstract

The COVID-19 pandemic has made access to face-to-face learning opportunities—the most common form of adult learning—impossible. Many firms have scaled back their training investments due to economic uncertainty. One way to fill these gaps is through self-directed learning via the Internet. Learning opportunities via apps and online videos are available flexibly in terms of time and location. But can online learning substitute for the lack of face-to-face courses, especially in the workplace where constant skill updating becomes ever more important? We wanted to know if online learning opportunities were used more in the first months of the pandemic, and if so, for which purposes and by which groups. Using data from the Adult Cohort of the German National Educational Panel Study (NEPS-SC6) and a supplementary web survey conducted in May and June 2020, we show that the work-related use of online learning was stronger in these months than before the crisis. At the same time, however, educational inequalities in the use of such opportunities were larger than before the pandemic. Thus, the expansion of online learning seems to benefit highly educated workers rather than educationally disadvantaged groups.

## Abstract (de)

Die COVID-19-Pandemie hat den Zugang zu Lerngelegenheiten in Präsenz – der häufigsten Form des Lernens im Erwachsenenalter – unmöglich gemacht. Viele Betriebe haben auf Grund der wirtschaftlichen Unsicherheit ihre Weiterbildungsinvestitionen heruntergefahren. Eine Möglichkeit, diese Lücken zu füllen, ist das selbstgesteuerte Lernen über das Internet. Lernangebote über Apps oder Online-Videos sind zeitlich und räumlich flexibel verfügbar. Aber können Onlineangebote das Lernen in Präsenz insbesondere in der Arbeitswelt, die von immer stärkeren Lernanforderungen geprägt ist, ersetzen? Wir wollten wissen, ob Online-Angebote in den ersten Monaten der Pandemie verstärkt genutzt wurden, und wenn ja, zu welchem Zweck und von welchen Gruppen. Mit den Daten der Erwachsenenkohorte des Nationalen Bildungspanels (NEPS-SC6) sowie einer Zusatzerhebung, die im Mai und Juni 2020 durchgeführt wurde, können wir zeigen, dass die berufliche Nutzung von Online-Lernangeboten während der ersten Monate tatsächlich stärker war. Gleichzeitig haben sich Bildungsungleichheiten in der Nutzung solcher Angebote jedoch verstärkt. Von der Ausweitung des Online-Lernens scheinen eher Hochgebildete zu profitieren und nicht „bildungsferne“ Gruppen.

## Introduction

The COVID-19 pandemic has triggered a sudden and powerful surge in digitalisation. Lifelong learning beyond initial education has gained importance due to the ongoing technological change in many areas of the working and everyday life already before the pandemic. During the first months of the pandemic, many people had to acquire new digital skills at a sudden. For example, communication was digitised in many workplaces, parents had to support their children in home schooling, and many services, such as booking medical appointments or buying clothes, could only be used online or via apps. Therefore, the pandemic increased the need for learning for many people in the short term, particularly in the digital domain (Ehlert et al. 2020; Janssen and Leber 2020).

Adult education is mostly conducted in non-formal training and informal learning,^1^ either in the context of paid work or organised by adult education centres (Autorengruppe Bildungsberichterstattung 2020; Ehlert 2017). The lion’s share of adult education, namely 75 per cent, is related to the workplace (Boeren et al. 2020). Before the COVID-19 pandemic, most of these learning opportunities were organised face-to-face (BMBF 2020a). However, in the first months of the pandemic, social distancing regulations intended to limit the spread of the coronavirus made it impossible to access face-to-face learning opportunities. Hence, adult education providers and centres, such as *Volkshochschulen*, were closed. Similarly, most companies could not offer the scheduled amount of further training, for example, due to economic uncertainty, because instructors were not available or because employees could not participate for private reasons (Bellmann et al. 2020). As a result, only about one third of the companies in Germany could realise or continue planned training courses by expanding or introducing online learning formats (Flake et al. 2020). Mostly, these were large firms (Kohl and Denzl 2020). With fewer face-to-face courses available in the professional and private sectors, those interested in adult education had to rely on alternatives.

One important alternative to face-to-face courses is online learning. According to the OECD (2020), *online learning* refers to the use of digital materials to support learning and does not necessarily take place at a distance but may be used in physical rooms to complement traditional forms of learning. But note that the term of online learning is not used uniformly in the literature (for a recent overview, cf. Singh and Thurman 2019). In our study, we use this term only in the context of distance learning via the Internet because this reflects the conditions of learning during the Covid-19 pandemic particularly well. New web-based learning formats, such as video tutorials, multimedia learning apps or wikis, have increasingly emerged for a few years. Online learning formats are available in all places with Internet connections and can be flexibly adapted to individual daily routines.

Data from the Adult Education Survey suggest that learning via the Internet was already common before the pandemic: in 2018, 46 per cent of the adults in Germany reported using the Internet frequently or very frequently to learn something new (Autorengruppe Bildungsberichterstattung 2020; Tab. H310web). However, there were only a few organised work-related online learning opportunities. According to data from PIAAC, only 12 per cent of all adults participated in online training, which is considerably below the OECD average of 20% (OECD 2020). According to data from the Adult Education Survey 2018, only 19 per cent of all adult education courses were at least partially designed as online courses, whereas pure online courses accounted for only 5 per cent in Germany (BMBF 2020a). Educational activities with digital media^2^ were realised most often among apprentices (87%), followed by unemployed persons (61%). In contrast, blue-collar workers participated rather rarely in this kind of courses (29%). Usage was particularly high among those groups of adults, whose work requires to use information from the Internet and/or who have a high level of decision autonomy, whereas in jobs with manual tasks participation was unlikely (BMBF 2020a, p. 43). Moreover, acquiring digital competencies is an important reason for participating in courses using digital media (BMBF 2020a, p. 28f).

Despite the general potentials and the diffusion of online learning formats in recent years, there is little empirical evidence of how the adult population used these opportunities in Germany or other industrialised countries during the COVID-19 pandemic. Adult education researchers fear that the pandemic has increased inequality in lifelong learning, as in many other areas, and has hit disadvantaged groups most. Educationally disadvantaged groups are expected to have had the fewest opportunities to learn online for various reasons (e.g., Boeren et al. 2020; James and Thériault 2020; Waller et al. 2020). On the one hand, these groups often have limited access to equipment or reliable Internet connection, or they might lack the basic digital skills necessary to engage in self-directed online learning (Boeren et al. 2020; OECD 2020). On the other hand, disadvantaged groups suddenly faced economic hardship more than others and had to put “earning before learning and (re)training” (James and Thériault 2020).

In this article, we contribute to the topic of inequalities in online learning by providing evidence on online learning during the first months of the COVID-19 pandemic in Germany.^3^ In the sense of an external shock, and regardless of all its grave consequences for health, economy, and social life, the pandemic provides the unique opportunity to investigate whether the sudden restriction of face-to-face courses has contributed to increased inequalities in learning or caused a compensating increase in online learning. We focus primarily on learning that is work-related because of its potential repercussions for employability and social inequality. Using data from the Adult Cohort of the German National Educational Panel Study (NEPS-SC6) collected before the pandemic and a supplementary web survey conducted in May and June 2020, we examine the following research questions:

- How prevalent was online learning in the first months of the pandemic? And in which context did individuals learn (for professional or personal reasons)?
- Do the socio-structural characteristics of participants in work-related online learning during the pandemic differ from the pre-pandemic period?
- How were pandemic-related changes in working conditions associated to online learning for professional reasons?

## Theoretical considerations

Previous research has impressively shown significant social inequalities in participation in adult education (e.g., Kilpi-Jakonen et al. 2015; James and Thériault 2020; Waller et al. 2020). One of the most important factors that favour participation is higher educational attainment. For work-related learning, which is the main focus of this study, this implies that participation increases with higher job requirements. Moreover, the resources that companies provide for further training significantly influence participation in adult education (Kleinert and Wölfel 2018).

Social inequalities in adult education participation are commonly explained by supply- and demand-side approaches (for an overview, cf. Becker 2018). Because nearly 75% of adult education takes place at the workplace (Boeren et al. 2020), employers’ decisions and support of further training and learning opportunities play a prominent role here. Companies offer training to invest in the productivity of their employees and, thus, to avoid the turnover of skilled workers. Human capital theory and labour market segmentation are often used to explain the resulting social inequalities in training participation. Accordingly, it is more highly educated workers with more demanding job tasks working in larger firms and higher positions, who are most likely to participate in adult education (Kleinert and Wölfel 2018).

On the supply side of workers, rational decision making and bounded rationality provide additional explanations of social inequalities in adult education. According to these approaches, different groups of workers possess different amounts of resources in terms of skills, time, and money. These disparities result in varying relative assessments of benefits, risks and costs of adult education. In addition, due to previous educational experiences, aspirations and motivation differ as well. These approaches are especially useful to explain participation in adult education programmes outside of the workplace, e.g. self-organised learning activities and investments in formal education and training, which often require cutting down working hours or quitting the job. Utilising online learning opportunities might be one of these forms of learning.

In the political discussion, the digital acquisition of knowledge in adulthood is seen as a central means of making lifelong learning accessible to a broad public and thus enabling adaptation to constant technological change (Rüber and Bol 2017). As a result, public policy has supported online learning for several years. Online learning formats are often associated with the hope that the pronounced and persistent social inequalities in adult education participation will dissolve due to diffused participation (e.g., OECD 2020). This presumption is grounded in the observation that the “digital divide” in Internet access declined substantially worldwide and especially in rich western countries such as Germany. With low-threshold access, online learning is presumed to reach societal groups who hardly participated in traditional forms of adult education organised as face-to-face events (BMBF 2020b). Thus, online learning is open to persons whose life situations, employers or jobs offer few learning opportunities. This implies that barriers and selectivity regarding the supply side of employers (and other adult education providers) are less important for online learning opportunities than face-to-face courses.

Nevertheless, compared to traditional face-to-face events, online learning is associated with some additional prerequisites on the demand side of potential learners. Appropriate technical equipment and a certain level of digital literacy are necessary: many adults use their mobile phones to access the Internet, often with limited data volumes (Boeren et al. 2020). While this “first order” of the “digital divide” is slowly but steadily decreasing, the so-called “second order digital divide”, which concerns differences in skills for Internet use, is still very relevant (Matzat and van Ingen 2020). As far as digital literacy is concerned, analyses from PIAAC show that—among the OECD countries—every sixth adult lacks even the most basic computer skills (OECD 2019). The necessary technical requirements are most likely to be met by workers in higher-paying occupations that involve computer use and demand for high digital problem-solving skills, and less so by workers in low-wage occupations that involve mostly manual tasks and hardly any demand for information and communication technology (ICT) skills (OECD 2020). Related to the latter, a younger age could positively impact participation because younger individuals are usually more used to digital technologies (Mauno et al. 2019) and easier to be reached by online offers (Boeren et al. 2020). Results from the Adult Education Survey 2018 confirm these inequalities among the workforce in Germany (BMBF 2020a). Thus, online learning may be part of the digital divide because not everybody uses the Internet for learning despite the wide-ranging availability. This may be one reason for digital inequality (Matzat and van Ingen 2020).

Moreover, online learning partly requires similar prerequisites as face-to-face courses, such as cognitive and self-organising abilities, time resources, career aspirations and motivation. Pandemic-related burden, such as overtime or unpaid care work, should therefore negatively impact online learning. Overall, the varying degrees to which online learning opportunities are used are strongly related to individual and professional resources. Nevertheless, learning online is a low-stake decision, which is less costly in terms of money and time than face-to-face learning. Hence, these prerequisites might have a lower effect on learning online, and therefore reduce social inequalities. Against the background that face-to-face training events were non-existent during the first lockdown in Germany and training needs increased abruptly, particularly in the digital sphere, we expect that online learning became highly prevalent among adult workers. In addition, we expect that due to increased demand, especially among lower skilled workers and occupations with a low degree of digitalisation before the pandemic, educational inequalities in online learning should be less pronounced during the pandemic than before. Thus, the pandemic may have bridged parts of the digital divide in terms of learning.

The COVID-19 pandemic has altered the work and family life of different groups of workers in very different ways. Hence, resources and barriers to learning differ strongly among potential online learners. For example, workers who had to reduce their working hours due to short-time work or layoffs had on average more time for digital learning. However, these changes often affected workers in occupations with relatively low job requirements, who may also sometimes have lacked the individual resources to learn in a virtual environment. Furthermore, given a higher workload and family caregiving responsibilities, we presume that essential workers and those with younger children might have had less time for online learning than before. Finally, we expect telework to be positively associated with online learning for two reasons: the sudden necessity of working remotely often made it necessary to learn new skills. Additionally, individuals who worked remotely usually had the required technical equipment available at home and possessed the necessary digital skills, either because their employers provided them or because they invested themselves.

## Data and methods

### Data and sample

To test these assumptions, we use data on the working population based on the NEPS Adult Cohort (NEPS-SC6), which includes education and employment biographies of more than 17,000 persons born between 1944 and 1986, who were living in Germany at the time of sampling (for details, cf. Allmendinger et al. 2019; Blossfeld et al. 2011). NEPS-SC6 comprises three subsamples drawn randomly from registers of a representative sample of German municipalities. The first subsample of persons born in 1956–1986 was interviewed for the first time in 2007/08 in the context of the IAB study ALWA (*Arbeiten und Lernen im Wandel*, [Working and learning in a changing world]; Kleinert et al. 2011). In 2009/10, this group was interviewed a second time in the context of NEPS. Additionally, the sample was refreshed and enhanced by older birth cohorts (1944–1955). A second refreshment of the sample took place in 2011. After the first interview, the participants have been followed up annually in a mixed-mode design (computer-assisted telephone and face-to-face interviews).

Additionally to the regular survey waves, a short supplementary web survey for NEPS-SC6 respondents dedicated to the COVID-19 pandemic took place in spring 2020. The survey was fielded from 15 May to 22 June, i.e. at the end of the first lockdown, which was put into effect end of March. At this time, retail slowly re-opened, but schools, day-care centres, restaurants and other public facilities were still largely closed. In the web survey, respondents were, among other things, asked questions on their employment situation and their participation in online learning during the lockdown.

In this article, we use data from the 11th Scientific Use File (SUF) of the NEPS-SC6 (10.5157/NEPS:SC6:11.1.0), which contains data from all survey waves up to the year 2018/19 plus the supplementary web survey dedicated to the COVID-19 pandemic (pTargetCORONA). Additionally, we use data from the latest regular survey wave (wave 12) of NEPS-SC6 (consortial data B145_C1), which took place shortly before the outbreak of the pandemic, between September 2019 and March 2020. For our analyses, we restricted the sample to respondents who participated in wave 12 and the Corona web survey. Thus, we observed each individual twice, once before the pandemic and once after the outbreak in late spring 2020. Moreover, we excluded respondents who had not been working before the pandemic, in February 2020. The final analysis sample consists of 1799 individuals.

Since all respondents in our sample come from a long-running panel survey subject to selective initial nonresponse and attrition, all descriptive results are weighted. These weights, which were specifically built for employed participants in the Corona web survey, account for the sample design of the NEPS-SC6 and adjust for panel attrition, using a rich set of information available in the data. In addition, the weights were post-stratified, i.e., the observed distributions were adjusted to the distribution of the target population in the official statistics (German Microcensus, statistics from 2019). For this calibration, year of birth, gender, country of origin, federal state, urbanisation, level of education and employment status were considered (for details on selectivity and weighting, cf. Würbach et al. 2021).

### Measures and analytic strategy

To examine the use of online learning opportunities, we estimated logistic regression models on the probability of using online learning opportunities for professional reasons. We present the results in the form of average marginal effects (AME). AME represent the change in probability of participating in online learning for professional reasons at the mean of all observations in our analysis sample in percentage points. To shed light on the degree of confounding between different explanatory variables, particularly education and ICT skills on the one hand and working conditions during the COVID-19 pandemic, on the other hand, we estimated a nested set of models.

As a dependent variable, we used information on online learning opportunities included annually in each regular NEPS wave since 2018/19. The question text reads: *“Since the last interview in […] have you used learning opportunities on the Internet or via apps (e.g. wikis, online forums, podcasts or YouTube) to learn professionally or privately?” *For the supplementary Corona web survey, this question was re-formulated to capture the change in the use of online learning in the first months of the pandemic: “*Since the start of the Corona crisis, have you used learning opportunities on the Internet or via apps (e.g., wikis, online forums, podcasts or YouTube) to learn professionally or privately?” *Thus, the question refers to a significantly shorter reference period than before, namely a maximum of three months compared to about one year in the last regular wave. In addition, in both the annual NEPS survey and the supplementary Corona web survey, respondents were asked whether these learning opportunities were used mainly for professional reasons, mainly for private interest or for both.

As the dependent variable in both surveys refers to different periods of use, it is not feasible to directly compare the likelihood of online learning in both time points in a joint regression model. Likewise, examining group differences in participation using interaction effects between period and group indicators would lead to an erroneous interpretation in the probability of participation. For this reason, we estimated separate models for the 2019/20 survey and for the Corona web survey to compare the likelihood of online learning during the first months of the pandemic with the situation before. All models are based on unweighted data to prevent very small respondent groups, which were given a higher weight, from distorting the standard errors. Possible selectivity bias due to non-response was instead controlled for by including those variables in the model which were also used for weighting. By estimating Wald tests, we additionally tested whether the effects of influencing factors differed statistically significantly from each other in the two survey waves considered. In this way, we assess more cautiously whether the association between participation in online learning and individual determinants differed before and during the COVID-19 pandemic.

We examine possible selectivity in online learning among working adults by including various indicators to distinguish relevant social groups, such as age, gender and family composition, migration background, region of residence (East or West Germany) and educational attainment. However, due to the small sample size in the lowest education group, we only distinguish between academics and non-academics (tertiary education). Moreover, we examine differences for respondents who said they performed only basic ICT tasks in their jobs compared to those with advanced ICT tasks in the job.

Table 1 shows the distribution of time-constant covariates in our analysis sample. In the last regular NEPS survey wave in 2019/20, respondents were between 34 and 76 years old, with a mean age of nearly 50 years. Almost one quarter of the sample either had immigrated to Germany themselves, or at least one of their parents had. 19 per cent lived in East Germany at the time of the last regular interview. 28 per cent cohabitated with children under 14 years of age, including more men (15%) than women (13%). The vast majority had a vocational educational and training (VET) degree (71%), less than 15 per cent had a tertiary degree, and another 15 per cent had no post-school degree. 65 per cent said they performed basic ICT tasks in their current job.

**Table 1:** Description of the sample (wave 2019/2020)

	Mean/%
*Age, in years*	49.7
*Migration background (1st and 2nd generation)*	23.1
*Living in East Germany*	18.8
*Men living without children under 14 years*	37.0
*Women living without children under 14 years*	35.1
*Men living with children under 14 years*	15.0
*Women living with children under 14 years*	12.9
*No post-school degree*	14.8
*Vocational education and training*	70.7
*Tertiary education*	14.5
*Basic ICT tasks in current job*	64.9

Source: NEPS-SC6, own analysis, weighted data, *N* = 1799

Additionally, we assessed the working situation at both time points. We use two dummy variables indicating essential work and telework as well as a categorical indicator on altered working hours. Table 2 shows the working situation in our analysis sample before and during the Corona crisis (weighted summary statistics). For nearly half of the respondents, working hours did not change during the first months of the pandemic. 14 per cent said they worked more than before, 38 per cent had less work or did not work at all during this period. Before the outbreak of the pandemic, the amount of work was more stable and working hours were less often reduced. When comparing the two surveys, the difference in the reference period has to be considered. In the 2019/20 survey, the question on changes in working hours covered the entire period since the previous interview (which on average had been conducted one year earlier). Conversely, the Corona web survey only asked about changes in a time span of three to four months. Almost one-third of respondents reported having the option to work remotely during the first months of the pandemic, compared to only four before the outbreak of the pandemic. Lastly, in the Corona web survey, 37 per cent said they worked in an essential occupation. No such information is available for the previous wave.

**Table 2:** Work situation before and during the Corona crisis (in %)

	2019/20	May/June 2020
*No changes in working hours*	55.3	47.8
*Reduced working hours/no work at all*	22.0	38.3
*Increased working hours*	22.7	13.9
*Working remotely*	3.9	33.2
*Essential work*^*a*^	–	37.3

Source: NEPS-SC6, own analyses, weighted data, *N* = 1799

^a^Only asked for in the supplementary Corona web survey

## Results

### Use of online learning opportunities before and during the pandemic

Before the outbreak of the pandemic, one-third of the respondents had used at least one online learning opportunity in the previous year. In contrast, in the first months of the pandemic, the share was one quarter. Due to the different reference periods mentioned before, the comparison of these percentages should not be interpreted as a decline in usage. Extrapolating the reference period of the supplementary Corona web survey from 3–4 months to one year might even suggest that online learning increased during the first lockdown.

**Fig. 1:**
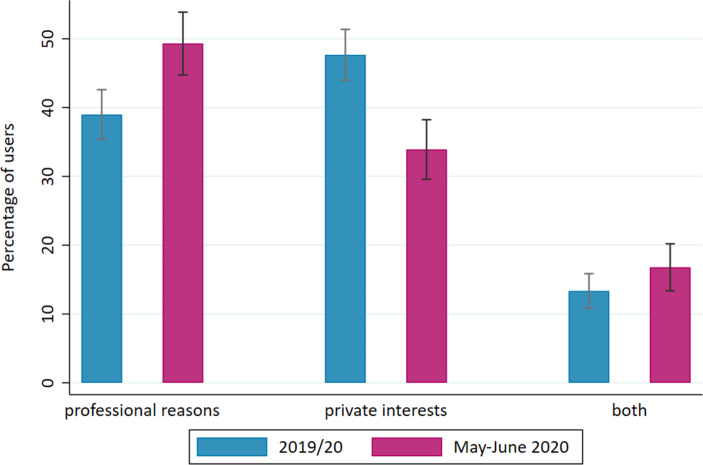
Reasons for online learning. (Notes: The confidence intervals indicate the uncertainty of the estimated utilisation rates. In 95% of cases, a comparable sample would produce a result in this range. For 24 persons (1.3%), no values on the use of online learning opportunities were available from the 2019/20 wave). (Source: NEPS-SC6, own analyses, weighted data. N = 1799)

Fig. 1 illustrates the increased importance of online learning for professional reasons during the first months of the pandemic. Before the pandemic, online learning was most frequently used for purely private interests (48%), second to purely professional reasons (39%), and seldom for both reasons (13%). During the first months of the pandemic, almost half of the workers who used online learning opportunities did so for purely professional reasons (49%). The group with mixed use increased slightly as well (17%). Conversely, online learning opportunities were used less often for purely private interests (34%). Overall, learning via the Internet and apps thus seems to have intensified in the first months of the pandemic, especially for professional purposes. To investigate the drivers of this change, we only examine online learning (solely or additionally) for professional reasons in further analyses.

### Group differences in online learning for professional reasons

Table 3 presents the results from the nested logistic regression models based on the period before the COVID-19 pandemic (Models 1a to 3a) and the first months of the pandemic (Models 1b to 3b). Models 1a/b contain socio-demographic variables and education. Hence they are used to estimate effects of those supply-side workers resources connected with educational attainment, migration background, region of residence, gender, and family obligations, which might affect usage of online learning opportunities constantly over time. Models 2a/b also include ICT tasks in the job and, therefore, additionally control for those resources, which are an important prerequisite for online learning. Models 3a/b additionally control the working situation at both time points, and hence take demand-side barriers to digital learning into account, which might have changed during the pandemic. Table 3 reports also Wald tests for effect size differences in the two sub-samples for each modelling step.^4^

**Table 3:** Probability of online learning before and during the COVID-19 pandemic (logistic regressions, average marginal effects, Wald tests of differences)

	M1			M2			M3		
	(a)	(b)		(a)	(b)		(a)	(b)	
	Before	During	Diff	Before	During	Diff	Before	During	Diff
Women w/o children *(ref. men w/o children)*	−0.02	0.01	0.03	−0.01	0.01	0.02	−0.01	0.01	0.02
	(0.02)	(0.02)	(0.37)	(0.02)	(0.02)	(0.47)	(0.02)	(0.02)	(0.44)
Men w children < 14	0.04	−0.01	−0.05	0.03	−0.01	−0.04	0.04	−0.01	−0.05
	(0.04)	(0.03)	(0.31)	(0.04)	(0.03)	(0.34)	(0.04)	(0.03)	(0.28)
Women w children < 14	0.00	−0.03	−0.03	0.02	−0.02	−0.04	0.02	−0.03	−0.05
	(0.04)	(0.03)	(0.55)	(0.04)	(0.03)	(0.46)	(0.04)	(0.03)	(0.38)
Age (in years)	−0.00^+^	−0.00	0.00	−0.00^+^	−0.00	0.00	−0.00^+^	−0.00	0.00
	(0.00)	(0.00)	(0.59)	(0.00)	(0.00)	(0.61)	(0.00)	(0.00)	(0.48)
Living in East Germany *(d)*	0.01	0.03	0.02	0.01	0.03	0.02	0.01	0.04^+^	0.03
	(0.03)	(0.02)	(0.52)	(0.03)	(0.02)	(0.53)	(0.03)	(0.02)	(0.49)
Migration background *(d)*	0.04+	0.01	−0.03	0.04	0.01	−0.03	0.04	0.01	−0.03
	(0.03)	(0.02)	(0.36)	(0.03)	(0.02)	(0.37)	(0.03)	(0.02)	(0.35)
Tertiary education *(d)*	0.07***	0.13***	0.06*	0.07***	0.13***	0.06*	0.06***	0.10***	0.04
	(0.02)	(0.02)	0.03	(0.02)	(0.02)	(0.03)	(0.02)	(0.02)	(0.38)
ICT: basic tasks in job *(ref. advanced ICT tasks)*	–	–	–	−0.06**	−0.03	0.03	−0.06**	−0.02	0.04
	–	–	–	(0.02)	(0.02)	(0.23)	(0.02)	(0.02)	(0.14)
ICT: no information	–	–	–	−0.05	−0.05	0.00	−0.02	−0.06	−0.04
	–	–	–	(0.04)	(0.04)	(0.96)	(0.05)	(0.04)	(0.92)
Essential work *(d)*	–	–	–	–	–	–	0.01	0.02	0.01
	–	–	–	–	–	–	(0.02)	(0.02)	(0.55)
Reduced working hours *(ref. unchanged)*	–	–	–	–	–	–	0.03	0.02	−0.01
	–	–	–	–	–	–	(0.02)	(0.02)	(0.64)
Increased working hours	–	–	–	–	–	–	0.04	0.04^+^	0.00
	–	–	–	–	–	–	(0.03)	(0.02)	(0.87)
Working hours: no info	–	–	–	–	–	–	−0.05	–	–
	–	–	–	–	–	–	(0.05)	–	–
Telework *(d)*	–	–	–	–	–	–	0.07^+^	0.09***	0.02
	–	–	–	–	–	–	(0.04)	(0.02)	(0.69)
Chi^2^	31.04**	58.37**	–	41.90**	62.49**	–	49.40**	88.47**	–
Pseudo R^2^	0.02	0.04	–	0.02	0.04	–	0.03	0.06	–

Source: NEPS-SC6, own analyses, unweighted data, *N* = 1775

*ref.* for reference categories, *d* for dummy variables, standard errors in brackets

^+^ *p* < 0.10, * *p* < 0.05, ** *p* < 0.01, *** *p* < 0.001

#### Gender and parenthood

According to our theoretical expectations, we assumed that persons with younger children in the household, and mothers with care responsibilities in particular, would have fewer chances to participate in online learning for professional reasons during the COVID-19 pandemic than before (Fuchs-Schündeln and Stephan 2020; Hipp and Bünning 2021; Zoch et al. 2021a). However, the results show that the effects of gender and children on online learning are not significantly different from zero when controlling for socio-demographic characteristics, neither before nor during the pandemic (Models 1a and 1b). Note, however, that the effects show the expected signs in Model 1b: in the first months of the pandemic, women (and men) with younger children were, in tendency, less likely to learn digitally than the reference group, men without children. In contrast, before the pandemic women with children were—similar to men with children—even more likely to learn than childless men. This picture does not change when controlling additionally for ICT tasks in the job (Models 2a and 2b) and for working conditions (Models 3a and 3b). Thus, although gender and family responsibilities often strongly correlate with working conditions, especially during the pandemic (Bünning et al. 2020; Zoch et al. 2021b), they seem to have little independent influence on work-related online learning. At the same time, these results are based on a small subsample and therefore require additional research.

#### Education

Theoretically, we expected a levelling effect between educational groups due to the sudden surge in digitalisation during the pandemic. Contrary to our expectation, our results suggest that the educational differences in the use of online learning for professional reasons were stronger during the COVID-19 pandemic than before. When controlling only for socio-demographic characteristics, the difference in the likelihood of usage between academics and non-academics increased from 7 percentage points before the pandemic to 13 percentage points during the pandemic (Models 1a and 1b in Table 3). This difference of six percentage points is statistically significant at the 5% level. This difference persists when controlling for ICT tasks in the job (Models 2a and 2b). When also considering the working situation, the effect of educational attainment on work-related online learning becomes slightly smaller, and the difference between the two time points is no longer statistically significant (Models 3a and 3b). In sum, these results suggest a polarisation by education in the use of online learning opportunities: the COVID-19 pandemic deepened the digital divide in terms of Internet usage for work-related learning. This polarisation seems partly related to compositional differences of workers and their resources and partly related to the different ways in which jobs were affected by the Corona crisis.

#### Use of ICT tasks in the job

One reason for the increase in work-related online learning during the pandemic might be that learning disproportionally grew in occupations that were already strongly characterised by computer use before the pandemic. In such occupations, highly qualified workers are usually overrepresented (Kirchner 2015). Before the COVID-19 pandemic, workers with basic ICT tasks were less likely to use online learning opportunities than workers with advanced ICT tasks (Model 2a). However, during the pandemic the effect of ICT tasks did not increase but became slightly smaller and lost statistical significance (Model 2b). The difference in effects before and during the crises is not statistically significant, so we cannot attribute this difference to the pandemic. Nevertheless, the change in effects might indicate a diffusion of online learning into occupations with low ICT affinity. Moreover, it is interesting to note that including ICT tasks in the model did not change educational disparities (Model 2 versus Model 1 in Table 3). Thus, the polarisation between educational groups reported above was not related to pre-pandemic ICT tasks use. Instead, the academically educated workers make greater use of online learning opportunities than the non-academically educated, regardless of the ICT tasks use in their job.

#### Working situation

Our results show that working conditions are one of the important explanators for participation in online learning in general. But in contrast to our theoretical assumptions, the results do not show any significant changes in the influence of working conditions on the use of online learning for professional reasons. Being in a job that was seen as “essential” during the pandemic played little role in participation—neither before nor during the COVID-19 pandemic. Both negative and positive changes in working hours were associated with a higher learning probability at both time points, compared to stability in work volume, but effects did not reach significance. Conversely, teleworkers were significantly more likely to participate in online learning than workers without the option to work remotely, particularly during the first months of the pandemic. Although telework was mainly used by highly educated workers (Kleinert et al. 2020), it is associated with a 9 percentage-point increase in the probability of online learning, even after controlling for educational attainment (Model 3b). Before the pandemic, online learning was already positively associated with telework (Model 3a). Thus, the pandemic has hardly brought any changes here. However, it is important to note that the group of persons who could work remotely increased significantly in the first months of the pandemic, from below 4 to 33 per cent (Table 2). Hence, the societal advantages of teleworking with regard to online learning opportunities grew strongly in relevance during the first months of the pandemic.

## Conclusions

By exploiting panel data from Germany, this study provides first insights into the short-term consequences of the COVID-19 pandemic regarding online learning of working adults. Data from the NEPS supplementary web survey conducted in May and June 2020 highlights that online learning played an important role during the pandemic. Online learning opportunities via the Internet and apps were used proportionally more often during the pandemic than before, particularly for professional reasons. On the one hand, this could be due to the greater need to learn how to work in digital contexts. At the onset of the pandemic, many workplaces were equipped with digital communication and information infrastructure and co-working tools to which workers had to adapt quickly. On the other hand, adult education and training could not be provided in the usual face-to-face format at that time. Both aspects are likely to have increased professional use of online learning. Additional data from the NEPS, collected at later stages of the pandemic, will be needed to disentangle the impact of these two factors.

Moreover, the educational inequality in participation in online learning seems to have increased in the first months of the pandemic. Even after controlling for further socio-demographic factors and associated resources, we observe a polarisation in online learning between lower and high educated workers. This trend is partly related to educational inequalities in the transformation of the working conditions brought about by the COVID-19 pandemic. Furthermore, our results suggest that the pandemic has not significantly changed the importance of the workplace conditions studied—working in an essential occupation, changes in working hours and telework—for the use of online learning opportunities. However, the fact that working conditions themselves changed abruptly and significantly for most workers in Germany in the first lockdown meant that online learning for professional reasons was subject to major change. In particular, high-educated workers were much more likely to work remotely than lower-educated workers (Kleinert et al. 2020). Employees who were able to work remotely were more likely than others to participate in online learning even before the pandemic, but in the first months of the pandemic, the group of teleworkers has grown sharply. The same tends to be true for persons with altered working hours.

Overall, our results do not confirm the presumption that an expansion of online learning opportunities would reduce social inequality in adult education participation. During the first months of the pandemic, the rise in online learning did not fully reach those groups that are generally less likely to participate in further training. Although online learning opportunities are presumed to be low-threshold, the digitisation of learning has not led to greater participation by lower educated, who are not only less likely to participate in further training but also less likely to have unlimited access to a reliable Internet connection as a prerequisite for online learning (Boeren et al. 2020; James and Thériault 2020). Instead, our results seem to confirm a “Matthew effect”, according to which already highly educated adults are those with the highest participation rates in adult education (Boeren et al. 2020). Thus, the COVID-19 pandemic seems to have widened the digital divide. Whether this inequality in usage of the Internet for learning has long-term consequences in terms of social inequality through differences in skill development and earnings should be the subject of future research.

In contrast, the fact that more online learning took place in jobs with only basic ICT tasks during the first lockdown gives some reason for optimism. It remains an empirical question, though, whether this is only a short-term effect related to workplaces, which were quickly equipped with digital communication and co-working infrastructure, or whether this group of workers has continued to learn online and will do so in the long run. Upcoming waves of the NEPS Adult Cohort may show how this trend will develop in the future.

In sum, our study provides first insights into the development of online adult education under pandemic conditions. Nevertheless, it also has some limitations. First, the use of online learning was surveyed by a single item only. Although we can describe the compensation of cancelled face-to-face learning by products on the Internet, we know little about the intensity, duration or topics of online learning. Second, the relatively small sample of NEPS-SC6 respondents who participated in the supplementary Corona web survey reduces the explanatory power of our results, as weak effects might not be detected. Moreover, the supplementary Corona survey was a web survey, which was taken by a selective group of participants who were possibly more digitally oriented than others. Despite trying to reduce selectivity by using weights for the descriptive results and by directly controlling for the known sources of selectivity in the multivariate analysis, we cannot completely rule out the possibility that this group reacted differently to the pandemic than other parts of the employed population in Germany, and hence the changes in and effects on online learning are under- or overestimated.

Despite these limitations, our study is one of the first to show empirical evidence on the topic of online learning during the pandemic. Our results suggest that more efforts are required on the part of policymakers and social partners to enable disadvantaged groups in the labour market, particularly low-skilled and unemployed workers, to participate in online learning and make digital skills acquisition a permanent feature of lifelong learning in the post-pandemic time. According to the OECD’s list of recommendations to policymakers (OECD 2020), it is important to develop basic digital skills in all groups of the population, find strategies to activate online learners, expand the range of online courses to reach also blue-collar workers, and ensure a reliable and fast digital infrastructure across the country.

## Footnotes

1 Non-formal training refers to non-institutionalised, but organised forms of learning, such as courses provided by employers, private training providers or professional organisations. Informal learning, in contrast, is neither institutionalised nor organised, but refers to conscious and unconscious processes of self-learning. Examples are learning by doing, learning from colleagues and supervisors, visiting congresses, or reading professional literature (Fialho et al. 2019; OECD 2020).

2 This term comprises hybrid and online formal and nonformal adult education, where the Internet is used to exchange materials and/or to communicate (BMBF 2020a). Hence, this accounts for far more educational activities than online courses.

3 An earlier version of this paper was published in German language as LIfBi Working Paper 94: 10.5157/LIfBi:WP94:1.0.

4 In robustness checks, we also controlled for occupational areas (KldB 2010, 1‑digit code). The results show similar patterns to the results shown in Models 3a and 3b. However, we refrained from showing these results because they might mask the effects of pandemic-induced changes in working conditions.
